# Exploring the Effective Components and Mechanism of Action of Japanese *Ardisia* in the Treatment of Autoimmune Hepatitis Based on Network Pharmacology and Experimental Verification

**DOI:** 10.3390/ph15121457

**Published:** 2022-11-24

**Authors:** Tian Fu, Yifei Chen, Junkui Li, Peili Zhu, Huajuan He, Wei Zhang, Ken Kin Lam Yung, Wei Wu

**Affiliations:** 1 School of Pharmacy, Guilin Medical University, Guilin 541199, China; 2 School of Traditional Chinese Medicine, Southern Medical University, Guangzhou 510515, China; 3 Department of Biology, Hong Kong Baptist University, Hong Kong 999077, China; 4 Golden Meditech Centre for NeuroRegeneration Sciences (GCNS), Hong Kong Baptist University, Kowloon Tong, Kowloon, Hong Kong 999077, China

**Keywords:** Japanese *Ardisia*, autoimmune hepatitis, network pharmacology, molecular docking, underlying mechanism

## Abstract

Japanese *Ardisia* is widely used as a hepatoprotective and anti-inflammatory agent in China. However, the active ingredients in Japanese *Ardisia* and their potential mechanisms of action in the treatment of autoimmune hepatitis (AIH) are unknown. The pharmacodynamic substance and mechanism of action of Japanese *Ardisia* in the treatment of AIH were investigated using network pharmacology and molecular docking technology in this study. Following that, the effects of Japanese *Ardisia* were evaluated using the concanavalin A (Con A)-induced acute liver injury rat model. The active ingredients and targets of Japanese *Ardisia* were searched using the Traditional Chinese Medicine Systems Pharmacology database, and hepatitis-related therapeutic targets were identified through GeneCards and Online Mendelian Inheritance in Man databases. A compound–target network was then constructed using Cytoscape software, and enrichment analysis was performed using gene ontology (GO) and Kyoto Encyclopedia of Genes and Genomes (KEGG) databases. Molecular docking technology was used to simulate the docking of key targets, and the AIH rat model was used to validate the expression of key targets. Nineteen active chemical components and 143 key target genes were identified. GO enrichment analysis revealed that the treatment of AIH with Japanese *Ardisia* mainly involved DNA–binding transcription factor binding, RNA polymerase II-specific DNA transcription factor binding, cytokine receptor binding, receptor-ligand activity, ubiquitin-like protein ligase binding, and cytokine activity. In the KEGG enrichment analysis, 165 pathways were identified, including the lipid and atherosclerotic pathway, IL-17 signaling pathway, TNF signaling pathway, hepatitis B pathway, and the AGE–RAGE signaling pathway in diabetic complications. These pathways may be the key to effective AIH treatment with Japanese *Ardisia*. Molecular docking showed that quercetin and kaempferol have good binding to *AKT1*, *IL6*, *VEGFA*, and *CASP3*. Animal experiments demonstrated that Japanese *Ardisia* could increase the expression of *AKT1* and decrease the expression of *CASP3* protein, as well as *IL-6*, in rat liver tissues. This study identified multiple molecular targets and pathways for Japanese *Ardisia* in the treatment of AIH. At the same time, the effectiveness of Japanese *Ardisia* in treating AIH was verified by animal experiments.

## 1. Introduction

Autoimmune hepatitis (AIH) is a common liver disease worldwide, seen in both men and women, but predominantly in women. According to epidemiological surveys, the incidence of AIH ranges from 0.67 to 2.0/100,000 people per year, and the prevalence ranges from 4.0 to 42.9/100,000 [1,2]. AIH has become the second most common inflammatory liver disease after viral hepatitis [3].

The pathogenesis of AIH is not completely understood. AIH is thought to be caused by genetic factors, molecular mimetic mechanisms, immune damage, and a variety of physical and chemical factors [4,5,6,7,8,9,10,11]. Prednisolone in combination with or without azathioprine (AZA) is generally recommended as the first-line drug for AIH [12], and second-generation alternatives, such as budesonide and tacrolimus, are recommended for this category of non-responders or intolerant patients, but these drugs have certain side effects. Traditional Chinese medicine has become increasingly important in the treatment of the disease in recent years.

Japanese *Ardisia*, known as *Ardisia japonica* or marlberry, is used as a medicinal plant in traditional Chinese medicine. It grows very slowly, and its leaves have a similar appearance to tea leaves. Bright red berries appear under the leaves in autumn, and therefore, it is also called ‘aidicha’ or ‘yedizhu’ in Chinese. Japanese *Ardisia* is mainly grown in the southern provinces of China, such as Hunan and Guangxi, where it is a popular medicinal herb used in Chinese folk medicine. The Chinese ancient medicine book ‘Compendium of Materia Medica’ records that *A. japonica* has the effect of ‘detoxification and promoting blood circulation’. The pharmacodynamic components of *A. japonica* are saponins, coumarins, benzoquinones, and flavonoids [13,14,15]. It has pharmacological activities, such as relieving cough and asthma, protecting the liver, and anti-inflammatory, anti-viral, and anti-tumor activities [16,17]. In clinical practice, *A japonica* is commonly used to treat chronic bronchitis, pulmonary tuberculosis, tuberculous pleurisy, and acute icteric hepatitis. Meanwhile, *A. japonica* has shown remarkable curative effects in the treatment of chronic hepatitis [14]. However, its pharmacodynamic ingredients and mechanism of action remain unclear.

**Figure 1:**
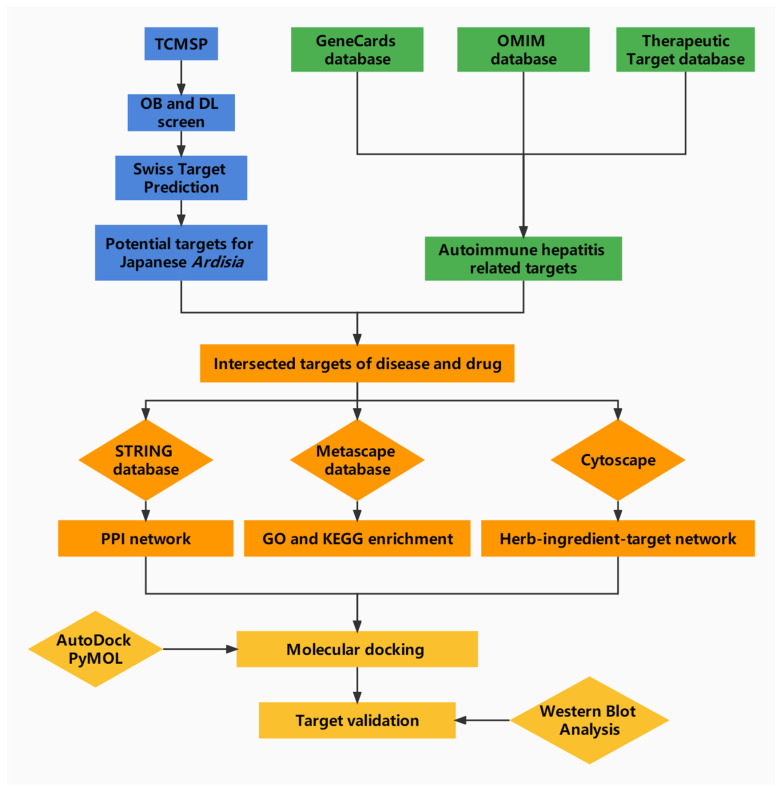
Network pharmacology analysis workflow.

Cyberpharmacology can effectively reveal the material basis and mechanism of action of Chinese medicine by systematically and integrally exploring the relationship between drugs and diseases [18]. Thus, the aim of this study is to systematically elucidate the mechanism of Japanese *Ardisia* in the treatment of AIH through network pharmacology and molecular docking analysis of the interaction between drug molecules and AIH-related targets, and to provide a theoretical basis for clinical research. The specific flow chart is shown in Figure 1.

## 2. Results

### 2.1. Screening of Active Compounds

Oral bioavailability (OB) is the fraction of an orally administered drug that reaches systemic circulation. This is an important consideration for bioactive molecules used as therapeutic agents. Drug likeness (DL) qualitatively assesses the capacity of a molecule to become an orally administered drug based on its bioavailability [19]. The main active components of Japanese *Ardisia* were obtained by searching the TCMSP database. Nineteen molecules with OB ≥ 30% and DL ≥ 0.18 were identified as bioactive compounds [20], as shown in Table 1.

### 2.2. Compound Target Interaction Network

**Figure 2:**
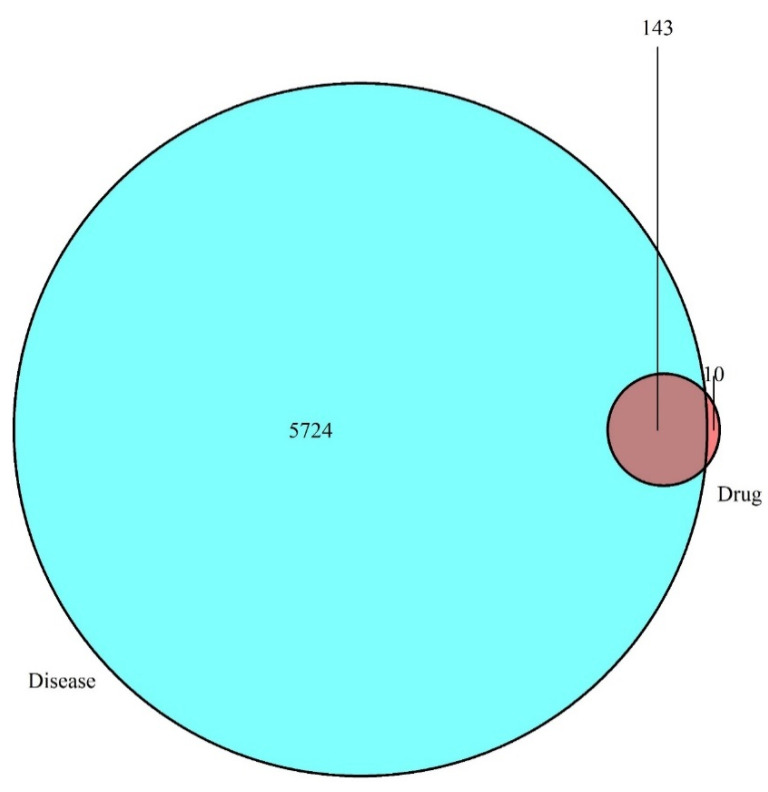
Intersection of drug targets and disease targets. Note: blue represents disease targets; red represents drug targets.

The information on the main bioactive components and corresponding targets in Japanese *Ardisia* were obtained from the TCMSP database (TCMSP, https://lsp.nwu.edu.cn/tcmsp.php, accessed on 14 November 2021). After screening to remove invalid gene IDs, the targets downloaded from the GeneCards (http://www.genecards.org, accessed on 14 November 2021), OMIM databases (OMIM, http://www.omim.org, accessed on 14 November 2021), and TTD (http://db.idrblab, accessed on 14 November 2021) were crossed with those from the TCMSP database to obtain potential targets for the treatment of AIH in Japanese *Ardisia*. The obtained data are presented as a Venn diagram (Figure 2). There were 5724 autoimmune hepatitis gene targets, of which 153 were potential targets related to the drug Japanese *Ardisia*. The drug-related genes and disease-specific targets were analyzed, and 143 key target genes were identified. The targets of the active ingredients of Japanese *Ardisia* are shown in Table 2.

### 2.3. Core Genes of the PPI Network

**Figure 3:**
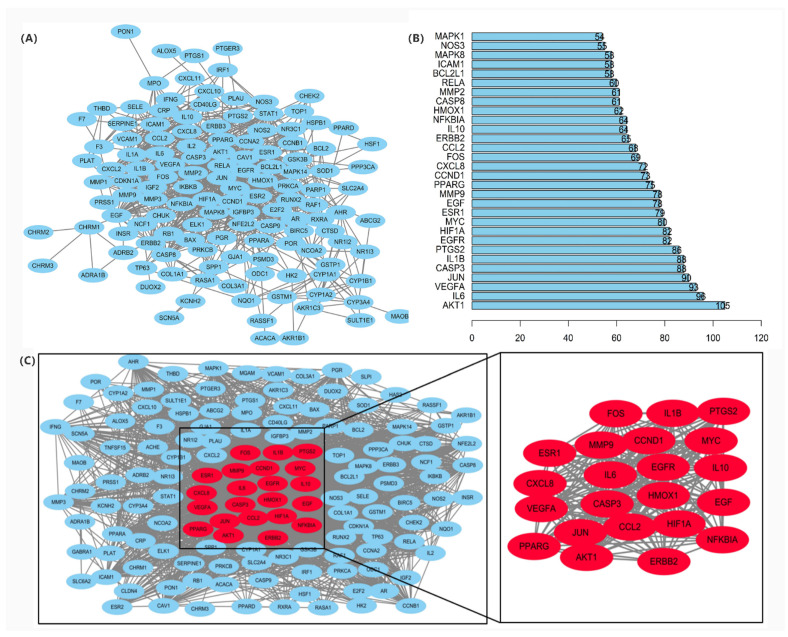
(**A**) PPI protein interaction network. Note: Network nodes represent proteins. Each node represents all proteins produced by a single protein-coding locus; the edge represents the protein-protein binding and promotes sharing function. (**B**) PPI network core genes. (**C**) Key targets of PPI network in the treatment of autoimmune hepatitis.

The common targets of Japanese *Ardisia* and autoimmune hepatitis were imported into the STRING protein interaction database (https://string-db.org, accessed on 15 November 2021) to construct the PPI network (Figure 3A). The top 30 target proteins analyzed by R software (https://www.r-project.org/ accessed on 15 November 2021) are shown in Figure 3B. The intersections of differential genes between Japanese *Ardisia* active ingredient targets and autoimmune hepatitis disease targets were imported into Cytoscape 3.7.0 (http://www.cytoscape.org, accessed on 15 November 2021) for topological analysis. The key targets were sorted according to the degree value, with higher degree values indicating nodes that were more central in the network and more important. Through the PPI protein interaction network, the disease-related targets are found, and the degree value is greater than the median by screening twice to find key targets. Thus, the key target genes for autoimmune hepatitis treatment mainly included *AKT1*, *IL6*, *VEGFA*, *CASP3*, *JUN*, *MYC*, etc. (Figure 3C).

### 2.4. Network Pharmacology Visualization of Japanese Ardisia

**Figure 4:**
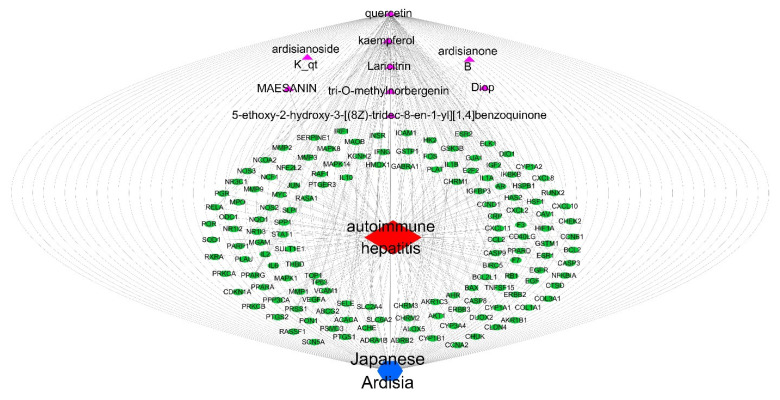
Network diagram of “Japanese *Ardisia*-component-gene-autoimmune hepatitis”. Note: Japanese *Ardisia* is blue; components are purple; the gene is green; and autoimmune hepatitis is red.

The target PPI information obtained from the STRING protein interaction database was imported into Cytoscape 3.7.0 software (http://www.cytoscape.org, accessed on 15 November 2021), and the common targets between Japanese *Ardisia*, its active components, and autoimmune hepatitis were visualized. The data were merged into a component-target network diagram via the merge function in Cytoscape 3.7.0 to obtain the network diagram of ‘Japanese *Ardisia*–component–gene–autoimmune hepatitis’ (Figure 4).

### 2.5. GO Functional Enrichment Analysis

**Figure 5:**
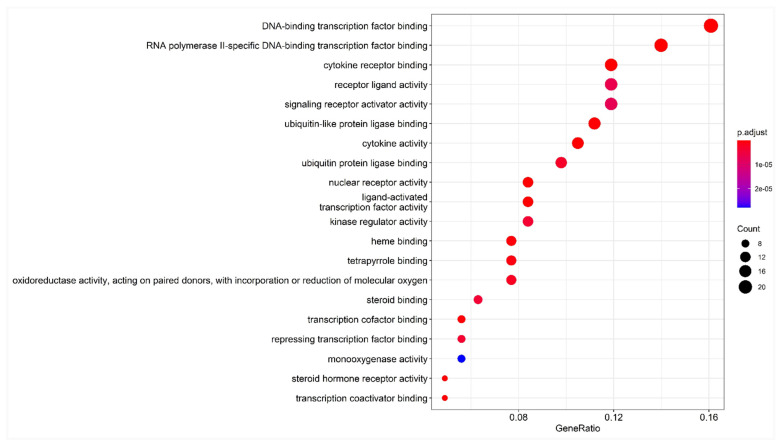
Bubble chart of GO enrichment analysis. Note: the abscissa is the gene ratio, the ordinate is the enriched pathway, the size of the point represents the number of genes, and the color represents the level of *p*-value.

The 20 GO nodes with the greatest number of annotated proteins were selected for display. These nodes mainly involved DNA–binding transcription factor binding, RNA polymerase II-specific DNA binding, cytokine receptor binding, receptor-ligand activity, ubiquitin-like protein ligase binding, cytokine activity, ubiquitin–protein ligase binding, nuclear receptor activity, ligand-activated transcription factor activity, and kinase regulatory activity. The *p*-values were arranged from largest to smallest, and visual analysis was performed using an advanced bubble graph (Figure 5). DNA–binding transcription factor binding had the most obvious effect, and the greatest number of genes, followed by RNA polymerase II-specific DNA binding, cytokine receptor binding, receptor-ligand activity, ubiquitin-like protein ligase binding, cytokine activity, and ubiquitin–protein ligase binding. Meanwhile, nuclear receptor activity, ligand-activated transcription factor activity, kinase regulator activity, and other pathways indirectly affected a series of signaling pathways, eventually causing changes in biological processes.

### 2.6. KEGG Pathway Enrichment Analysis 

**Figure 6:**
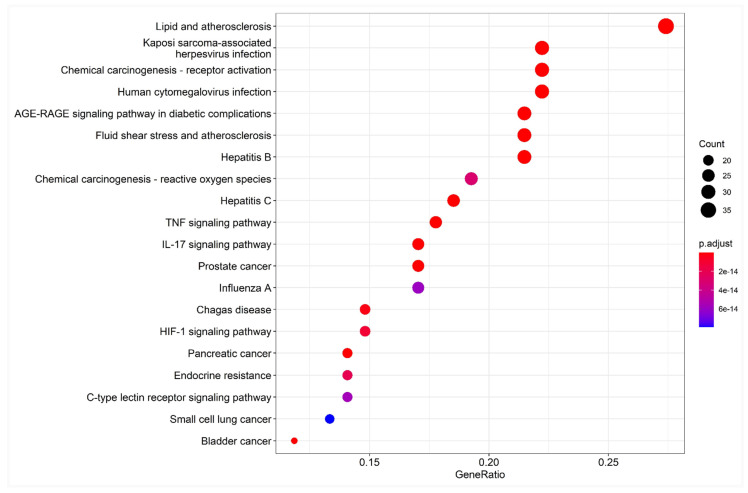
Bubble chart of KEGG pathway enrichment analysis. Note: the abscissa is the gene ratio, the ordinate is the enriched pathway, the size of the point represents the number of genes, and the color represents the level of *p*-value.

One hundred and sixty-three pathways were identified in the KEGG enrichment analysis. The top 20 signaling pathways mainly involved the lipid and atherosclerosis pathway, Kaposi sarcoma-associated pathway, human cytomegalovirus infection pathway, IL-17 signaling pathway, TNF signaling pathway, hepatitis B pathway, and the AGE-RAGE signaling pathway were involved in diabetic complications (Figure 6). The results suggest that Japanese *Ardisia* can be used to treat autoimmune hepatitis through multi-target and multi-pathway regulation.

### 2.7. Molecular Docking

**Figure 7:**
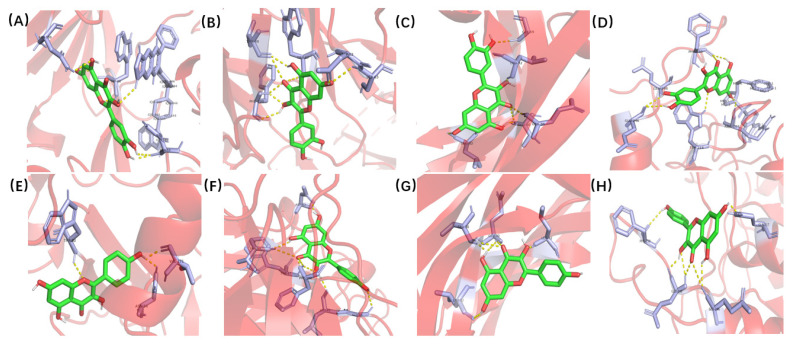
Molecular docking of quercetin and kaempferol with *AKT1, IL6, VEGFA* and *CASP3.* (**A**) Quercetin and *AKT1*. (**B**) Quercetin and *IL6*. (**C**) Quercetin and *VEGFA*. (**D**) Quercetin and *CASP3*. (**E**) Kaempferol and *AKT1*. (**F**) Kaempferol and *IL6*. (**G**) Kaempferol and *VEGFA*. (**H**) Kaempferol and *CASP3*.

The X-ray crystal structures of the target molecules *AKT1*, *IL6*, *VEGFA*, and *CASP3* were obtained from the PDB protein structure database. PyMOL 2.5 was then used to remove water molecules and small molecules with ligand affinity. Subsequently, the protein receptor and ligand files were converted into PDBQT format using AutoDock Tools 1.5.6. AutoDock Vina 1.1.2 was used to characterize the molecular docking and calculate its affinity. The conformation with the highest affinity was selected as the final docking conformation, and PyMOL (https://pymol.org/2/ accessed on 16 November 2021) and AutoDock software (https://autodock.scripps.edu/ accessed on 16 November 2021) were used to visualize the docking results in the form of two-dimensional and three-dimensional diagrams (Figure 7). If the binding energy between the molecule and the target protein is negative, the ligand and receptor can spontaneously bind, and if the binding energy is less than −5 kcal/mol, a stable docking structure can be formed [21]. The docking binding energies between quercetin and *AKT1*, *IL6*, *VEGFA*, and *CASP3* were −4.91, −5.75, −4.86, and −5.89 kcal/mol, respectively. The docking binding energies between kaempferol and *AKT1*, *IL6*, *VEGFA*, and *CASP3* were −5.6, −6.42, −5.04, and −5.12 kcal/mol, respectively. The details are shown in Table 3. The Japanese *Ardisia* active ingredients quercetin and kaempferol had a good binding ability with the four key targets.

### 2.8. Animal Experiments

#### 2.8.1. Validation of the Therapeutic Effectiveness of Japanese *Ardisia*

**Figure 8:**
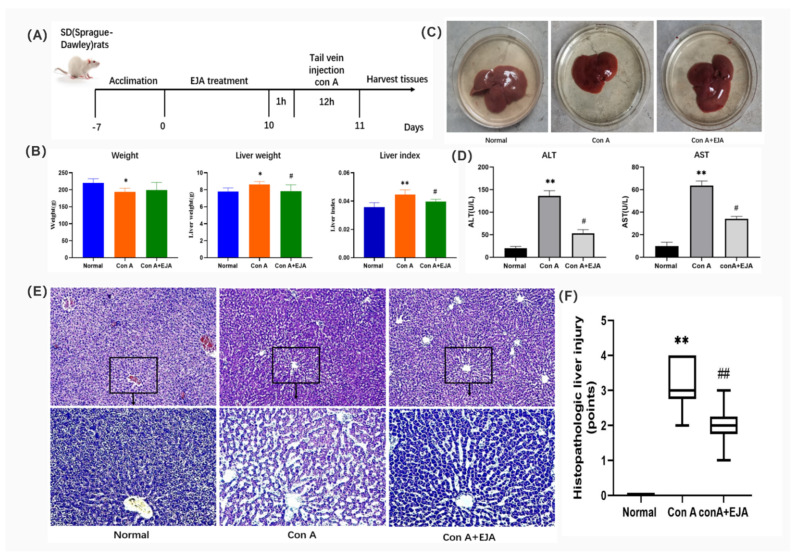
(**A**) Description of the rat model of acute immune liver injury used in this study. (**B**) Body weight, liver weight, and liver coefficient changes in rats 10 days after EJA treatment. Compared with normal group, ** p < 0.01, * p < 0.05; Compared with model group, ^#^ *p* < 0.05. Note: EJA is an extract of Japanese *Ardisia.* Data were shown as mean ± SEM (*n* = 6 per group). (**C**) Rat liver treated with EJA. (**D**) Effect of EJA on serum ALT and AST levels. Compared with normal group, ** p < 0.01; Compared with model group, ^#^ *p* < 0.05. Note: EJA: an extract of Japanese *Ardisia.* Data were shown as mean ± SEM (*n* = 3 per group). (**E**) Histopathological sections of rat liver (HE staining, 200× 400×). (**F**) Effect of Japanese *Ardisia* pretreatment on the scoring of liver pathological sections of rats with con A-induced liver injury. Compared with normal group, ** p < 0.01; compared with model group, ^##^ *p* < 0.01. Note: EJA is an extract of Japanese *Ardisia.* Data were shown as mean ± SEM (*n* = 6 per group). Scoring criteria: 0—no necrosis; 1—individual cell necrosis; 2—less than 30% necrosis; 3—30–60% necrosis; 4—greater than 60% necrosis [22].

To investigate the efficacy of Japanese *Ardisia* in the treatment of AIH, we established a Con A-induced immunological liver injury model (Figure 8A). Firstly, we found that Con A caused changes in body weight, liver weight, and liver coefficients in rats after ten days, with some reversal effect after preadministration of Japanese *Ardisia* (Figure 8B). The rats’ liver tissue was then taken out and checked for cholestasis. Con A group showed signs of cholestasis and inflammation, and there was some relief from cholestasis after the drug was administered (Figure 8C). By testing serum markers of liver injury (ALT and AST), the results showed that Con A-induced acute liver injury resulted in a significant increase in ALT and AST levels. In contrast, preadministration of Japanese *Ardisia* was able to alleviate the altered biochemical levels. (Figure 8D).

Furthermore, HE staining of liver tissues showed that the model group had a large infiltration of inflammatory cells and a large amount of vacuolar-like degeneration of hepatocytes compared to the control group. Liver tissue damage was alleviated after preadministration of Japanese *Ardisia* (Figure 8E,F), indicating that the pre-administration of the drug was able to slow down the con A-induced histopathological damage to the liver.

#### 2.8.2. The Effect of Japanese *Ardisia* on *AKT1*, *CASP3*, and *IL-6* Protein Levels

**Figure 9:**
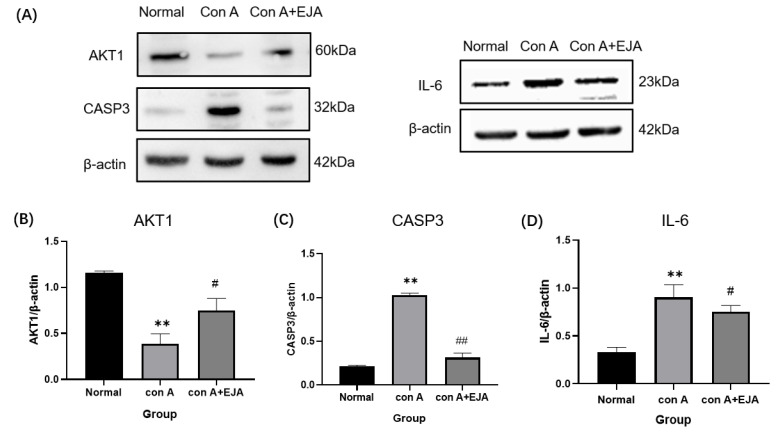
Western blot analyses. (**A**) Western blot analysis of *AKT1, CASP3,* and *IL-6*. (**B**–**D**) Relative expression of *CASP3, AKT1,* and *IL-6* in the liver tissue of SD rats. Compared with normal group, ** p < 0.01; Compared with model group, ^##^ *p* < 0.05, ^#^ *p* < 0.05. Data were shown as mean ± SEM (*n* = 3 per group). Note: EJA is an extract of Japanese *Ardisia*.

Western blot analyses are shown in Figure 9, which revealed that *CASP3* and *IL-6* protein expression levels were significantly higher (*p <* 0.01), and *AKT1* expression levels were significantly lower (*p <* 0.01) in the model group compared to the normal control group. *CASP3* protein expression was significantly lower (*p <* 0.01), *AKT1* protein expression was significantly higher (*p <* 0.05), and *IL-6* protein expression was significantly lower (*p <* 0.05) in the Japanese *Ardisia* administration group after 10 days compared to the model group.

## 3. Discussion

AIH is a chronic inflammatory disease characterized by abnormally high levels of serum autoantibodies, hypergammaglobulinemia, and serum transaminases [23]. With the advancement of medical technology in recent years, AIH has received widespread attention and has gradually become a research hotspot.

Chinese medicine’s multi-component and multi-target properties, and thus its holistic and systemic action characteristics, make it unique in its advantages and potential for complex diseases, but this complexity has also limited its application and development.

Cyberpharmacology analyzes drug action at the systemic level and reveals the synergistic mechanism of drug action on the human body, which fits well with the dialectical and holistic view of Chinese medicine theory, and it is expected to bring a breakthrough to Chinese medicine research characterized by a holistic approach, providing new methodological support for Chinese medicine to move from empirical to theoretical science [24,25,26]. We used network pharmacology to create a component-shared target network map in this study to systematically reveal the material basis and molecular mechanism of Japanese *Ardisia* for the treatment of AIH.

This study provides key information about the anti-hepatitis effect of Japanese *Ardisia*. The TCMSP database analysis resulted in the screening of 19 active ingredients. 143 effective targets for the treatment of autoimmune diseases were identified using databases, such as GeneCards and OMIM. According to the results of the component–target network analysis, 150 target genes were associated with the drug-disease target intersection. The core genes were *AKT1*, *IL6*, *VEGFA*, and *CASP3*. *AKT1*, also known as protein kinase B, regulates a wide variety of cellular functions, including cell proliferation, survival, metabolism, and angiogenesis, in both normal and malignant cells [27]. *IL6* is a pro-inflammatory cytokine with a wide variety of biological functions. It is involved in physiological activities such as the inflammatory response, cellular immunity, and hematopoietic regulation. *IL6* plays major roles in the differentiation of B cells into immunoglobulin-secreting cells and antibody production, the activation of T cell proliferation and differentiation, the immune response, and the promotion of inflammatory reactions [28]. *IL6* is rapidly synthesized in response to tissue injury or an inflammatory infection. This promotes the body’s defense function by stimulating the acute immune response and the hematopoietic system. When the tissue recovers its homeostasis, *IL6* synthesis is discontinued [29]. The continuous activation of the *IL6* pathway is associated with liver injury and hepatocellular carcinoma [30]. *VEGFA* induces endothelial cell proliferation, promotes cell migration, inhibits apoptosis, and induces the permeabilization of blood vessels. It is essential for both physiological and pathological angiogenesis. *CASP3* is a cysteine aspartate protease that participates in the activation cascade of cysteine proteases. *CASP3* plays an important role in inflammation and tumor progression [31]. It is highly expressed in patients with hepatitis B [32], and it regulates cell proliferation and apoptosis [33] and tumor invasion and metastasis [34].

Japanese *Ardisia* has a therapeutic effect on hepatitis via a mechanism that may be related to key molecules involved in the regulation of autoimmune hepatitis. It inhibits associated inflammatory factors by regulating various signaling pathways, downregulating the concentrations of serum hyaluronic acid and tumor necrosis factor, protecting hepatocytes from injury, reducing liver inflammation, and protecting against lipid peroxidation [35]. Inflammatory stimulation is the cause of many chronic diseases [36]. GO enrichment analysis showed that the anti-hepatitis effect of Japanese *Ardisia* is related to the inflammatory response, cell cycle regulation, and hormone metabolism. In the KEGG enrichment analysis, 163 pathways were identified, including the lipid and atherosclerosis pathway, IL-17 signaling pathway, TNF signaling pathway, human cytomegalovirus infection pathway, fluid shear stress and atherosclerosis pathway, hepatitis B pathway, and AGE–RAGE signaling pathway in diabetic complications. It can be seen that the pathway is mainly related to oxidative stress, immune regulation, and inflammatory responses, with the AGE-RAGE signaling pathway being closely related to inflammation, which activates the MAPK and NF-KB pathways and interferes with immune and oxidative stress responses [37]. TNF is a key regulator of the inflammatory response, and its receptors TNFR1 and TNFR2 activate complex signaling pathways that lead to a series of inflammatory responses in the vascular endothelium, including thrombosis, leukocyte adhesion, and vascular leakage [38]. The IL-17 signaling pathway is involved in neutrophil infiltration and inflammatory responses and can be restricted by the ACE2 downregulation of the STAT3 pathway, thereby slowing down neutrophil infiltration and inflammation. These pathways may be the key to effective autoimmune hepatitis treatment with Japanese *Ardisia*, which affects the secretion of certain substances by acting on specific receptors or enzymes, to achieve its therapeutic effect.

The binding interaction between each compound and its receptor was scored by the molecular docking program. A lower score indicated more stable binding between the ligand and its receptor [39,40]. Through the molecular docking of quercetin and kaempferol, the effective components of Japanese *Ardisia*, with the key targets *AKT1*, *IL6*, *VEGFA*, and *CASP3*, the docking binding energy and the number of intermolecular hydrogen bonds were obtained. The results showed that quercetin and kaempferol had a good binding ability with the key targets and can spontaneously bind to form a stable binding conformation.

Animal studies have shown that Japanese *Ardisia* is effective in the treatment of AIH. Western blot analysis revealed that Japanese *Ardisia* could reduce the expression of *CASP3* and *IL-6* while increasing the expression of *AKT1*. These proteins are involved in the AGE–RAGE signaling pathway in diabetic complications. According to this, the Japanese *Ardisia* may be able to modulate these key targets to achieve therapeutic effects on immune liver injury.

Additionally, there are also limitations to the research at this stage, as network pharmacology techniques can only predict drug composition and targets qualitatively. High-performance liquid chromatography (HPLC) or ultraviolet spectrophotometry (UV) should be used to determine the plausibility of the screened active ingredients, and this should be combined with pharmacology, pharmacodynamics, and pharmacokinetics to make the screened active ingredients and mechanism of action more convincing.

## 4. Materials and Methods

### 4.1. Databases

The Traditional Chinese Medicine Systems Pharmacology (TCMSP) database, an analysis platform (http://tcmspw.com/tcmsp.php (accessed on 14 November 2021)), GeneCards (http://www.genecards.org (accessed on 14 November 2021)), Online Mendelian Inheritance in Man (OMIM, http://www.omim.org (accessed on 14 November 2021)), the Therapeutic Target Database (TTD; http://db.idrblab), the Search Tool for Retrieval of Interacting Genes (STRING) protein interaction database (https://string-db.org (accessed on 15 November 2021)), Cytoscape 3.7.0 (http://www.cytoscape.org (accessed on 15 November 2021)), and the Protein Data Bank (PDB) protein structure database (https://www.rcsb.org/pdb (accessed on 16 November 2021)) were used in this study.

### 4.2. Screening of Active Ingredients of Japanese Ardisia

The components of Japanese *Ardisia* were searched in TCMSP (TCMSP, https://lsp.nwu.edu.cn/tcmsp.php, accessed on 14 November 2021) and screened for active compounds by oral bioavailability (OB) and drug similarity (DL), as well as potential targets of action for activity, the active compounds obtained from the screening are presented in the form of a list.

### 4.3. Screening of Target Diseases

GeneCards, OMIM, and TTD were used to identify hepatitis-related gene targets. Specific search parameters, such as the keyword ‘autoimmune hepatitis’, were used for data collection. Using R 4.0.4 software (https://www.r-project.org/ accessed on 15 November 2021) to remove duplicate regions of gene targets, the intersection of the active ingredient and disease target was obtained and plotted as a Venn diagram.

### 4.4. Target Protein Localization and Interaction Analysis

The cross-targets of Japanese *Ardisia* in autoimmune hepatitis treatment and prevention were imported into the STRING protein interaction database for analysis. The PPI network was mapped using the Cytoscape 3.7.0 software. A protein–protein interaction (PPI) network was constructed according to proximity centrality, intermediate centrality, and degree value, and the key targets were screened using Cytoscape 3.7.0. 

### 4.5. Construction of a Component-Target-Disease Interaction Network of Japanese Ardisia with Autoimmune Hepatitis

The previously acquired common genes and the associated active ingredients were visualized and analyzed using Cytoscape 3.7.0 software.

### 4.6. Gene Ontology and Kyoto Encyclopedia of Genes and Genomes Pathway Enrichment Analysis

The potential targets of Japanese *Ardisia* for autoimmune hepatitis treatment were imported into the Gene Ontology (GO) and Kyoto Encyclopedia of Genes and Genomes (KEGG) databases. GO was selected for enrichment analysis, KEGG was selected for pathway analysis, and the *p*-value threshold was set at <0.05 to determine the enrichment pathways of key targets. An advanced bubble diagram was constructed from the enrichment results: the smaller the *p*-value, the higher the enrichment; the larger the bubble, the richer the genes were.

### 4.7. Molecular Docking

The two-dimensional structures of quercetin, kaempferol, and laricitrin were obtained from the TCMSP database and saved as a Mol2 file. AKT serine/threonine kinase 1 (*AKT1*), interleukin 6 (*IL6*), vascular endothelial growth factor A (*VEGFA*), and caspase 3 (*CASP3*) were selected as the target proteins for molecular docking experiments. The three-dimensional structures of *AKT1*, *IL6*, *VEGFA*, and *CASP3* were downloaded from the PDB protein structure database and saved in PDB format [41]. Crystal structures with high resolution and corresponding bioactive ligand complexes were preferentially selected [42]. The structures of the protein macromolecules and small-molecule compounds were imported into AutoDock 4.2.6 software (https://autodock.scripps.edu/ accessed on 16 November 2021) for molecular docking, and the docking results were analyzed using the PyMOL (https://pymol.org/2/ accessed on 16 November 2021) visualization tool.

### 4.8. Animal Experiments

#### 4.8.1. Drug

The whole herb of Japanese *Ardisia* was ground into a coarse powder and kept in a dry, cool environment. A weighed amount of coarse powder was soaked in ten times the amount of water for one night before being decocted twice for 1.5 h each time. The decoctions were combined and concentrated to 1 g/mL (1 mL equals 1 g raw material), the concentrate was centrifuged at 5000 rpm for 15 min, the supernatant was collected and concentrated to 1.5 g/mL (1 mL equals 1.5 g raw material), and the obtained concentrate was refrigerated at 4 °C [43,44].

#### 4.8.2. Animal Grouping and Drug Administration

SPF-grade male SD (Sprague-Dawley) rats, weighing (200 ± 10) g, with six animals per cage were housed in specific pathogen-free facility with a 12 h light and 12 h dark cycle at 22 °C. All mice were randomly divided into three groups, control group (*n* = 6), con A group (*n* = 6), and extract of Japanese *Ardisia* (EJA) (*n* = 6) groups after being fed adaptively for a week. The control and model groups were given saline, while the administration group was given 36 g/kg of Japanese *Ardisia* by gavage once daily for 10 days. One hour after the last dose, all groups were given 25 mg/kg con A in the tail vein, except for the control group which was given an equal amount of saline in the tail vein, rats were euthanized 12 h after con A treatment, and their livers were dissected from the rats.

The kits purchased from Nanjing Jiancheng Institute of Biological Engineering were used, and the alanine transaminase (ALT) (Cat.No.C009-2-1) and aspartate transaminase (AST) (Cat.No.C010-1-1) measurements were performed according to the instructions provided by the kit supplier.

All the animals were provided by Hunan Sleek Jingda Laboratory Animal Co. License No. SCXK (Xiang) 2019-0005. Animal welfare and experimental procedures followed the regulations of the Animal Ethics Committee of Guilin Medical College.

#### 4.8.3. Histopathological Section Analysis of Liver

Tissue removed from 2.8.2 was fixed in 4% paraformaldehyde, paraffin sections were embedded, and, finally, the pathological sections were observed by HE staining, and the pathological changes of liver tissues were observed under an electron microscope.

#### 4.8.4. Western Blot

To verify the protein expression level, we extracted protein from rat liver tissue, weighed a certain amount of liver tissue, fully lysed it with RIPA lysis solution (Solarbio, Beijing, China), used SDS-PAGE electrophoresis, and then transferred it to PVDF (Solarbio, Beijing, China) membrane. We then blotted and closed them at room temperature for one hour, and then we incubated them with *AKT1* (60203-2, proteintech, Wuhan, China), *CASP3* (ab184787, Abcam, Cambridge, UK), *IL-6* (ab259341, Abcam, Cambridge, UK), β-actin antibody (66,009, proteintech, Wuhan, China), and incubated them overnight at 4 °C. Then, we incubated them with goat anti-rabbit IgG (H + L) and goat anti-mouse Ig G (H + L) for 1 h at room temperature. The protein bands were visualized using an ECL chemiluminescence kit (Beyotime, Shanghai, China) and quantified under Image J system, version 6.0.

### 4.9. Statistical Analysis

The experimental results are presented as mean ± SEM for each group, with at least three independent experiments. The differences between the treatment and normal groups were analyzed by a one-way analysis of variance using GraphPad Prism 8.0 (GraphPad Software Inc., San Diego, CA, USA). *p* < 0.05 was used to indicate statistical significance.

## 5. Conclusions

In summary, we first performed a network pharmacology and molecular docking approach to elucidate the anti-AIH effects and potential mechanisms of Japanese *Ardisia*, and finally determined the effect of Japanese *Ardisia* anti-AIH on the expression of key target proteins by Western blotting analysis. Japanese *Ardisia* tends to work through the IL-17 signaling pathway, the TNF signaling pathway, the AGE–RAGE signaling pathway in diabetic complications, and other targets such as *AKT1*, *IL-6*, and *CASP3* to exert drug effects. The potential signaling pathways uncovered in this study lay the theoretical groundwork and point the way for future experimental validation.

## Figures and Tables

**Table 1:** Basic information on the active compounds of Japanese *Ardisia*.

MOL ID	MOL Name	OB	DL
MOL010934	Ardisianoside K	31.98	0.63
MOL010953	Triterpenoid glycoside 1	34.11	0.63
MOL010964	Maesanin	42.77	0.35
MOL010973	Rapanone	34.15	0.24
MOL010974	Tri-O-methylnorbergenin	33.17	0.41
MOL010976	Triterpene glycoside 4	41.4	0.63
MOL010981	Triterpenoid glycoside 3	44.04	0.6
MOL010982	2,5-dihydroxy-3-[(10Z)-pentadec-10-en-1-yl][1,4] benzoquinone	34.74	0.6
MOL010983	2,5-Dihydroxy-3-[(10Z)-pentadec-10-en-1-yl] cyclohexa-2,5-diene-1,4-dione	37.3	0.32
MOL010985	2-hydroxy-5-methoxy-3-pentadecaenylbenzoquinone	41.61	0.32
MOL011002	5-ethoxy-2-hydroxy-3-[(10Z)-pentadec-10-en-1-yl][1,4] Benzoquinone	42.77	0.38
MOL011003	5-ethoxy-2-hydroxy-3-[(8Z)-tridec-8-en-1-yl][1,4] benzoquinone	43.23	0.3
MOL011019	Ardisianone A	44.22	0.25
MOL011020	Ardisianone B	60.9	0.2
MOL001663	(4aS,6aR,6aS,6bR,8aR,10R,12aR,14bS)-10-hydroxy-2,2,6a,6b,9,9,12a-heptamethyl-1,3,4,5,6,6a,7,8,8a,10,11,12,13,14b-tetradecahydropicene-4a-carboxylic acid	32.03	0.76
MOL002879	Diop	43.59	0.39
MOL000422	Kaempferol	41.88	0.24
MOL009278	Laricitrin	35.38	0.34
MOL000098	Quercetin	46.43	0.28

OB, oral bioavailability; DL, drug-likeness.

**Table 2:** Possible targets for each component.

MOL ID	Ingredients	Drug-Acting Targets of Disease
MOL010934	Ardisianoside K	NR3C1
MOL010964	Maesanin (C_23_H_36_O_4_)	ACHE
MOL010974	Tri-O-methylnorbergenin	PRSS1
MOL011003	5-ethoxy-2-hydroxy-3-[(8Z)-tridec-8-en-1-yl][1,4] benzoquinone	ACHE
MOL011020	Ardisianone B	GABRA1, NCOA2
MOL002879	Diop	CHRM3
MOL000422	Kaempferol	PTGS1, AR, PPARG, NCOA2, PRSS1, PGR, CHRM1, ACHE, CHRM2, GABRA1, F7, RELA, IKBKB, BCL2, AHSA1, CASP3, MAPK8, PPARG, CYP3A4, CYP1A1, ICAM1, SELE, VCAM1, CYP1B1, ALOX5, GSTP1, AHR, PSMD3, SLC2A4, NR1I3, DIO1, GSTM1, GSTM2, AKR1C3
MOL009278	Laricitrin	ESR1, AR, PPARG, ESR2, GSK3B, PRSS1, PTGS1, NCOA2
MOL000098	Quercetin	PTGS1, AR, PPARG, NCOA2, AKR1B1, PRSS1, F7, ACHE, GABRA1, RELA, EGFR, VEGFA, CCND1, BCL2, FOS, EIF6, CASP9, PLAU, RB1, IL6, AHSA1, CASP3, TP63, ELK1, NFKBIA, POR, CASP8, RAF1, PRKCA, HIF1A, RUNX1T1, ERBB2, PPARG, ACACA, CYP3A4, CAV1, MYC, CYP1A1, ICAM1, SELE, VCAM1, PTGER3, BIRC5, DUOX2, NOS3, HSPB1, MGAM, CYP1B1, CCNB1, ALOX5, GSTP1, NFE2L2, NQO1, PARP1, AHR, PSMD3, SLC2A4, COL3A1, DCAF5, NR1I3, CHEK2, HSF1, CRP, RUNX2, RASSF1, CTSD, IGFBP3, IGF2, IRF1, ERBB3, PON1, DIO1, NPEPPS, HK2, RASA1, GSTM1, GSTM2

**Table 3:** Docking and binding energy of main components of Japanese *Ardisia* and core targets (kcal·mol^−1^).

Target Molecules	*AKT1*	*IL6*	*VEGFA*	*CASP3*
MOL000098 (Quercetin)	−4.91	−5.75	−4.86	−5.89
MOL000422 (Kaempferol)	−5.6	−6.42	−5.04	−5.12

## Data Availability

All data generated or analyzed during this study are included in this published article.
